# ADVANCING EQUITY AND COMMUNITY ENGAGEMENT IN AGING IN CENTERS ON AGING

**DOI:** 10.1093/geroni/igae098.0725

**Published:** 2024-12-31

**Authors:** Joseph Gaugler, Robbin Frazier, Jane Mahoney

**Affiliations:** Chair; Co-Chair; Discussant

## Abstract

The ultimate goal of much gerontological scholarship is to improve the lives of older people. To meet the needs and preferences of our rapidly diversifying communities of older persons, creating equitable partnerships with older adults, families, neighborhoods, and organizations is often necessary, if not essential. This symposium aims to highlight centers/institutes on aging across the U.S. that have adopted various strategies and approaches to build partnerships with aging communities, co-create innovative scholarship, culturally adapt evidence-based/evidence-informed programs, and effectively disseminate knowledge to achieve health equity in aging. Our symposium will feature equity and community engagement missions, activities, opportunities, and challenges for four centers on aging, representing different types of institutions and areas of the country. Featured centers/institutes include the University of Texas-Austin’s Texas Aging & Longevity Consortium, Wayne State University’s Institute of Gerontology, Johns Hopkins University’s Center for Equity and Aging, and the University of Minnesota’s Center for Healthy Aging and Innovation. Jane Mahoney, MD, Director of the Community-Academic Aging Resource Network (CAARN) of the University of Wisconsin-Madison, will synthesize equity and community engagement efforts in centers/institutes on aging and will provide a roadmap for future initiatives to sustain this critical work.

